# Dyadic Dynamic in Generalized Political Attitudes: Homophily, Relationship Satisfaction, and Convergence

**DOI:** 10.5334/irsp.1102

**Published:** 2026-04-13

**Authors:** Andreas Miculka, Roland Imhoff

**Affiliations:** 1 Department of Social and Legal Psychology, University of Mainz, Germany

**Keywords:** conspiracy mentality, right-wing authoritarianism, social dominance orientation, response surface analysis, homophily

## Abstract

Despite the social nature of generalized political attitudes such as conspiracy mentality, right-wing authoritarianism (RWA), and social dominance orientation (SDO), dyadic phenomena concerning these attitudes have not been comprehensively investigated. Employing Response Surface Analysis on two longitudinal samples of couples in romantic relationships (*N*_1;T1_ = 249 couples, *N*_1;T2_ = 76, *N*_2;T1_ = 312, *N*_2;T2_ = 263), this research examined a) couple similarity in generalized political attitudes, b) whether similarity concurrently and longitudinally predicted relationship satisfaction, and c) whether couples converged in their attitudes over time. Partners exhibited substantial similarity in all attitude dimensions. However, there was only little support that similarity predicted relationship satisfaction. Finally, romantic partners did not converge in their attitudes over time, indicating that couple similarity reflected initial assortment rather than convergence. The wide prevalence of ideologically congruent relationships may restrict the range of political views that individuals are exposed to, potentially playing a role in political polarization.

## Introduction

Separating one’s political views from personal relationships can present a formidable challenge. This is especially the case when the beliefs in question are strongly held ideological views, such as *conspiracy beliefs*, the conviction that an event was caused by the secret cooperation of a group of people in pursuit of a malevolent goal (Bale 2007). A prominent example of this dynamic are the various conspiracy theories accompanying US president Donald Trumps’ political campaigns and time in office, which are reported to have led to conflicts and estrangement within many families (Phillips 2025). Systematic analyses of relatives and close friends of conspiracy believers have shown decreases in relationship satisfaction, perceived closeness, and the frequency and quality of interactions following the onset of such beliefs (Moskalenko, Burton & Bloom, 2023; Mousaw 2022). However, recent research suggests that it may not be the conspiracy belief itself, but the distance between the conspiracy belief of an individual and the corresponding belief of their loved one that causes the relationship to deteriorate. Toribio-Flórez et al. (2024) found that the negative influence of conspiracy beliefs on perceived relationship quality was moderated by the extent of the individual’s own conspiracy beliefs, thus implying that attitudinal distance could be responsible for the decrease in relationship satisfaction. This result echoes what Higgins et al. (2021) refer to as *shared reality*, the idea that humans seek a sense of perceived commonality in feelings and beliefs about the world. In line with this perspective, relationship partners generally align on a variety of attributes, a phenomenon known as *homophily* (Watson et al. 2004). However, no research has investigated whether this pattern holds for core ideological attitudes, such as *conspiracy mentality, right-wing authoritarianism* (RWA), and *social dominance orientation* (SDO). Thus, in this paper, we discuss a) to what extent couples are ideologically similar (i.e., their attitudinal distance is comparatively small), b) to what extent this similarity relates to relationship quality and satisfaction, and c) whether similarity between partners increases over time.

Previous research on the more general question of political homogeneity in romantic relationships has found that couples are highly similar regarding their left-right political orientation (Leikas et al. 2018), party identification (Lampard 1997), and specific political attitudes (Alford et al. 2011). While these are interesting and relevant findings, they also contain some limitations. For example, both members of a couple may consider themselves right-of center and vote for the same party, but her primary reason could be a desire for lower taxes while his motivation may be to decrease immigration. Indeed, research shows that individuals have different understandings of the left-right scale (Bauer et al. 2017) and that partisan affiliation is better understood as a social identity category rather than the agglomeration of ideological attitudes (Green, Palmquist & Schickler 2002). Therefore, the present research instead focuses on *generalized political attitudes*, stable ideological belief systems that predict specific attitudes towards attitude objects with a common trait (Imhoff & Bruder 2014). Of these, the ones with the greatest theoretical importance and research interest in social psychology are the two dimensions of Duckitt and Sibley’s (2016) dual-process model (RWA and SDO) as well as the recently increasingly researched conspiracy mentality (Imhoff, in press).

Duckitt and Sibley’s (2016) dual-process motivational model of ideology and prejudice distinguishes two ideological attitudes that express independent motivations and are accompanied by contrasting worldviews and personality traits. RWA refers to an individual’s preference for upholding the traditional norms and the cohesion of the ingroup through uncritical obedience towards its authorities and aggression towards individuals perceived to be deviant (Altemeyer 1981). RWA predicts a wide range of authoritarian phenomena, such as lower support for human rights, civil liberties, and democratic values (Cohrs et al. 2005) as well as higher support for severe punishment of criminals (Altemeyer 1996) and torture of prisoners of war (Larsson, Björklund & Bäckström 2012). In contrast, SDO describes an individual’s preference for group-based hierarchy and inequality (Ho et al. 2012). Similar to RWA, SDO is one of the most powerful predictors of prejudice (Sibley & Duckitt 2008). Moreover, SDO is related to support for various social policies that assert dominance over other groups, such as wars of aggression, harsh sentencing for criminals, the death penalty, and torture (Pratto et al. 1994; Sidanius et al. 2006), as well as opposition to policies that alleviate social inequality, such as affirmative action or social welfare (Gutiérrez & Unzueta 2013). Individuals high in SDO have also been found to more strongly value prestige in their career choices (Lepièce et al. 2016) and exhibit heightened competitive behavior (Cozzolino & Snyder 2008). While high values on both RWA and SDO are associated with right-wing political attitudes (van Assche et al. 2019), RWA more closely corresponds to a right-wing social orientation, while high SDO is predominantly associated with a right-wing economic orientation (Lönnqvist & Kivikangas 2019).

Conspiracy mentality, the general tendency to endorse conspiracy beliefs (Imhoff, in press), has more recently emerged as a particularly fruitful area of research. Conspiracy mentality has been found to be associated with prejudice towards high-power individuals (Imhoff & Bruder 2014), distrust of well-established scientific knowledge (van der Linden 2015), as well as criminal behavior (Jolley et al. 2019) and political violence (Imhoff, Dieterle & Lamberty 2021). Crucially, it is conceptually distinct from RWA and SDO and explains incremental variance in prejudice beyond their contributions (Imhoff & Bruder 2014), making the construct a valuable addition to the two aforementioned generalized political attitudes. In contrast to RWA and SDO, conspiracy mentality is also associated with extreme political views rather than specifically right-wing attitudes (Imhoff et al. 2022). While it is already known that people expect or perceive lower relationship satisfaction when their partner does not hold the same extent of conspiracy beliefs (Toribio-Flórez, Green & Douglas 2024), dyadic data showing the association with actual partner similarity is missing.

Despite their social nature and the plethora of research conducted on these attitudes, no study to date has comprehensively examined dyadic dynamic in conspiracy mentality, RWA, and SDO. Building on the aforementioned research, we hypothesized that the generalized political attitudes of romantic partners would exhibit a significant positive cross-partner correlation. In order to evaluate the magnitude of this effect, we also postulated that the similarity in generalized political attitudes would be equal to or larger than similarity in values. Secondly, we aimed to test the similarity-satisfaction link. There is some evidence that similarity can increase relationship satisfaction (Arránz Becker 2013; Mäenpää & Jalovaara 2014), but there are also null findings (Shiota & Levenson 2007; Watson et al. 2004). Some support for this hypothesis in the specific realm of conspiracy mentality was obtained in the recent research of Toribio-Flórez et al. (2024), who found that attitudinal distance negatively predicted relationship satisfaction. However, their research was only based on the self-report of one relationship partner, making it prone to various distortive influences. Lastly, we investigated partner convergence. Most prior research has thus far not found support for the convergence hypothesis in other personality domains (Alford et al. 2011; Humbad et al. 2010; Watson et al. 2004). Nevertheless, it has not been investigated within the domain of generalized political attitudes. If there is any effect, it has been supposed that partner convergence may mostly happen at the beginning of relationships (Alford et al. 2011), so we retained this prediction with the qualifier that the effect will be moderated by relationship duration.

### The present research

We conducted two studies, collecting data from partners of a romantic dyad at two measurement waves each. We estimated partner similarity (*Hypothesis 1*), as well as the association between similarity and relationship satisfaction. To overcome methodologically problematic limitations of past research, we used a state-of the art methodological approach, Dyadic Response Surface Analysis (DRSA; Schönbrodt et al. 2018), to test our hypothesis that higher partner similarity in generalized political attitudes a) concurrently and b) longitudinally predicts higher relationship quality (*Hypothesis 2*). Convergence was tested as an increase of dyadic similarity as a function of the moderator relationship duration, entailing the assumption that long-established relationships will feature less convergence over a set period of time compared to more recently established relationships (*Hypothesis 3*). Both studies were pre-registered.

## Study 1

### Methodology

#### Transparency and openness

All data, analysis code, and additional materials have been made available at https://osf.io/d8739. All studies’ hypotheses, designs, and analyses were pre-registered (OSF A). We report all manipulations, measures, and exclusions in all studies.

#### Participants and design

To address the formulated hypotheses, we conducted a quantitative longitudinal study with two measurement waves. Because of the uncertainties concerning the power of the DRSA and the extent of attrition between the measurement time points, no exact target sample size was defined prior to data collection (see OSF D1 for power considerations). The data were collected from January 14 to January 31 and March 12 to March 31, 2022. The study participants were recruited through the mailing list of the psychological institute of the university, social media, and online platforms. Participants and their partners provided informed consent and filled out a questionnaire on the online platform SoSci Survey,^1^ which was available in German and English. In total, 275 full dyads completed the questionnaire, of which 24 were excluded due to the preregistered careless responder analysis (speeding) and two due to a non-preregistered careless responder analysis (straightlining). The final data set at T1 consisted of 249 dyads. The age ranged from 18 to 78 years (*M* = 29.8 years, *SD* = 11.7 years) and the mean relationship duration was 6.6 years (*SD* = 8.9 years). The final data set at T2 consisted of 76 dyads. A detailed description of the sample composition can be found in the accompanying OSF project (OSF D2).

#### Measures

Table 1 summarizes the measures used in the study. Further information on the specific items and response scales can be found in the codebook (OSF C).

**Table 1:** Study measures.


CONSTRUCT	SCALE	AUTHORS	TRANSLATION

Conspiracy Mentality	Conspiracy Mentality Scale (CMS)	Imhoff and Bruder (2014)	Original Scale Authors

Right-Wing Authoritarianism	Authoritarianism Short Scale (KSA-3)	Beierlein et al. (2015)	Nießen et al. (2019)

Social Dominance Orientation	SDO_7(S)_ scale	Ho et al. (2015)	Aichholzer (2019), Six et al. (2001), Study Authors

Political Orientation	Left-Right Self-Placement scale	GESIS-Leibniz-Institut Für Sozialwissenschaften (2019)	Original Scale Authors

Relationship Quality	Revised Dyadic Adjustment Scale (RDAS)	Busby et al. (1995)	Study Authors

Values	Human Values Scale (HVS)	Schwartz et al. (2015)	Original Scale Authors


*Note*. In addition, participants were asked to provide their gender, age, highest attained level of education, as well as their country of residence. We also preregistered and measured vaccination against COVID-19 and relationship dissolution, but both variables displayed almost no variance and were thus not analyzed further.

#### Data analysis

Data analysis was performed using the programming language R. The scripts used to conduct data analysis can be found in the accompanying OSF project (OSF B).

**Hypothesis 1**. In order to assess this hypothesis, we calculated cross-partner correlation coefficients for conspiracy mentality, RWA, SDO, political orientation, and the two higher-order value dimensions of the HVS. We considered the dyads to be indistinguishable because the conducted empirical tests of distinguishability (Garcia & Kenny 2018) did not support the assertion that gender was a meaningful distinguishing variable for the collected data. As the ordering of the dyad members within each dyad is arbitrary in the indistinguishable case (Griffin & Gonzalez 1995), we used the intraclass correlation coefficient (*ICC*) rather than the Pearson correlation coefficient. In addition, we transformed each *ICC* into a *z* score using Fisher’s transformation in order to test whether the cross-partner correlation of a variable was higher than the average of the cross-partner correlations of the value dimensions (Griffin & Gonzalez 1995).

**Hypothesis 2**. Frequently, congruence hypotheses such as this one are tested using linear regression with the absolute difference between the partners’ attitudes as the independent variable and the dyad’s relationship quality as the dependent variable. While such usage of difference scores is a simple and intuitive method of examining congruence hypotheses, it is also methodically problematic because it entails implicit, untested assumptions (Peter et al. 1993). For instance, using difference scores as a predictor in a regression assumes that psychological effects of (dis-)similarity are equal on every level of the variable. More generally, it artificially collapses a three-dimensional relationship (between actor score, partner score, and outcome) into a two-dimensional relationship (between difference score and outcome) without examining whether placing such constraints on the data is warranted (Edwards 2002). Thus, we used DRSA (Schönbrodt, Humberg & Nestler 2018) in the present research. DRSA is based on a second-order polynomial regression model, which allows for linear and curvilinear main effects as well as an interaction effect for both dyad members’ scores. The regression parameters can then be used to calculate the *surface parameters a*_1_ through *a*_5_, which describe the surface of the variable’s relationships in the three-dimensional space of actor score, partner score, and outcome variable. The mathematical conditions that must be satisfied in order to support a congruence effect in the broad sense are: (1) *a*_3_ = 0, (2) *a*_4_ < 0, and (3) *a*_5_ = 0 (Humberg, Nestler & Back 2019). In addition, if one wants to rule out any main effects of the predictors (congruence effect in a strict sense), two additional conditions must be met: (4) *a*_1_ = 0 and (5) *a*_2_ = 0 (Humberg, Nestler & Back 2019).

We followed the procedure detailed by Schönbrodt et al. (2018) for the setup and execution of the DRSA. We applied the constraints for indistinguishable dyads, which are equal actor effects, equal partner effects, equal predictor means and variances, equal outcome intercepts, and equal residual variances (Olsen & Kenny 2006). Finally, parameter estimates, standard errors, and significance tests were obtained through a bootstrapping process with 10,000 iterations. The regression assumptions were tested using QQ-plots, descriptive statistics, the Durbin-Watson test, the White test, and generalized variance inflation factor scores to assess predictor multicollinearity.

**Hypothesis 3**. To assess this hypothesis, a linear mixed model was calculated for each of the difference scores. The predictors of each regression were measurement time point and relationship duration (measured at T1), while the criterion variable was the respective difference score. The model included a random intercept for each dyad. A negative main effect of measurement time point would indicate convergence, whereas a negative interaction term would suggest that relationship duration moderates the convergence effect in the hypothesized direction. The assumptions of the models were tested using plots, descriptive statistics, and the Durbin-Watson test.

### Results

#### Preparatory analyses

The anonymized data can be found in the accompanying OSF project (OSF C). The means, variances, reliabilities, and intercorrelations of all study variables at T1 are reported in Table 2. Analysis of the QQ-plots and descriptive statistics indicated that all study variables were approximately normally distributed (see OSF D3). A dropout analysis indicated that mainly high conspiracy mentality and preference for self-enhancement values predicted dropout in participants. On the dyad level, neither the difference scores of the study variables nor the mean dyad relationship quality predicted dropout.

**Table 2:** Means, variances, reliabilities, and intercorrelations of study variables.


	α	*M*	*SD*	1	2	3	4	5	6	7

**1.** CM	.91	3.63	1.18	(.85)						

**2.** RWA	.83	2.32	0.67	.30***	(.80)					

**3.** SDO	.76	2.74	1.00	.28***	.45***	(.78)				

**4.** POL	–	4.20	1.71	.22***	.43***	.53***	(.83)			

**5.** ST-SE	.67	1.26	1.18	–.10	–.25***	–.40***	–.35***	(.78)		

**6.** OP-CO	.66	0.68	1.11	.01	–.29***	–.14*	–.08	.03	(.77)	

**7.** RQ	.80	51.94	6.56	–.15*	–.15*	–.12	–.08	.21***	.11	(.81)


*Note. N*_T1_ind_ = 498. CM: Conspiracy mentality, RWA: Right-wing authoritarianism, SDO: Social dominance orientation, POL: Political orientation, ST-SE: Self-transcendence vs. self-enhancement, OP-CO: Openness to change vs. conservation, RQ: Relationship quality, α: Internal consistency (Cronbach’s alpha). Retest reliabilities are shown on the diagonal (*N*_T2_ind_ = 200). *p < .05, **p < .01, ***p < .001.

#### Hypothesis testing

**Partner Similarity (H1)**. *ICC*s of cross-partner similarity showed that couples displayed similarity above that expected by chance in all generalized political attitudes, as well as political orientation and values. The *ICC*s, 95% confidence intervals (*CI*), and significance tests are reported in Table 3.

**Table 3:** Cross-partner correlations and significance tests.


	*ICC*	95% *CI*	*F*(248, 249)	*p*

Conspiracy Mentality	.51	[.42, .60]	3.10	<.001

Right-Wing Authoritarianism	.41	[.30, .51]	2.40	<.001

Social Dominance Orientation	.33	[.21, .43]	1.97	<.001

Political Orientation	.49	[.39, .58]	2.96	<.001

Self-Transcendence vs. Self-Enhancement	.26	[.14, .37]	1.70	<.001

Openness to Change vs. Conservation	.22	[.10, .33]	1.56	<.001


*Note. N*_T1_ = 249. *ICC*: Intraclass correlation coefficient, *CI*: Confidence interval. Significance tests (*p* values) were adjusted for multiple comparisons using the Holm-Bonferroni correction.

Conspiracy mentality demonstrated significantly higher partner similarity than the mean of the value dimensions (*z* = 5.05, *p* < .001). Partners were also more similar in RWA than in values (*z* = 3.02, *p* = .005). However, similarity in SDO was not significantly different from similarity in values (*z* = 1.46, *p* = .145). In an exploratory analysis, we found that assortment on demographic variables did not substantially contribute to the observed couple similarity, as *ICC*s corrected for age and education were only marginally lower than the uncorrected *ICC*s (see OSF D4). In a non-uniformity analysis of the correlations using a procedure by Leikas et al. (2018), only RWA demonstrated significant non-uniformity, with interpretation of the regression slopes suggesting that the cross-partner correlation was approximately .20 at the low end of the RWA distribution (one standard deviation below the mean) and .60 at the high end (see OSF D5).

**Concurrent relationship quality (H2a)**. Four DRSA models were computed, one for each political attitude dimension predicting concurrent relationship quality. The polynomial regression and DRSA assumptions were mostly satisfied, with the exception of homoscedasticity for three models and independence of residuals for one model. However, as the final standard errors and *p* values were estimated via bootstrapping, these violations of the polynomial regression assumptions should not substantially impact the results (Efron & Tibshirani 1993). The regression and surface parameters of the DRSAs are presented in Table 4.

**Table 4:** Results of the DRSAs for concurrent relationship quality.


	CONSPIRACY MENTALITY	RWA	SDO	POLITICAL ORIENTATION

*b*_1_	–0.12** (0.05)	–0.13* (0.05)	–0.10* (0.05)	–0.05 (0.05)

*b*_2_	–0.08 (0.05)	–0.07 (0.05)	–0.09* (0.05)	–0.05 (0.05)

*b*_3_	0.08 (0.05)	–0.04 (0.04)	0.01 (0.04)	–0.03 (0.05)

*b*_4_	0.04 (0.08)	0.12 (0.08)	0.00 (0.06)	0.06 (0.09)

*b*_5_	0.01 (0.06)	–0.08 (0.04)	0.04 (0.03)	–0.02 (0.05)

*a*_1_	–0.21** (0.07)	–0.19* (0.09)	–0.20* (0.08)	–0.10 (0.08)

*a*_2_	0.12 (0.09)	0.01 (0.05)	0.05 (0.07)	0.01 (0.09)

*a*_3_	–0.04 (0.06)	–0.06 (0.05)	–0.01 (0.04)	0.00 (0.05)

*a*_4_	0.05 (0.16)	–0.23 (0.15)	0.05 (0.11)	–0.12 (0.17)

*a*_5_	0.06 (0.04)	0.04 (0.04)	–0.03 (0.03)	–0.01 (0.03)


*Note. N*_T1_ = 249. Standard errors in parentheses. Regression estimates can be interpreted as β weights due to prior standardization. *b*_1_: linear actor effect, *b*_2_: linear partner effect, *b*_3_: squared actor effect, *b*_4_: interaction effect, *b*_5_: squared partner effect, *a*_1_ = *b*_1_ + *b*_2_, *a*_2_ = *b*_3_ + *b*_4_ + *b*_5_, *a*_3_ = *b*_1_ – *b*_2_, *a*_4_ = *b*_3_ – *b*_4_ + *b*_5_, *a*_5_ = *b*_3_ – *b*_5_. *p < .05, **p < .01, ***p < .001.

**Figure 1:**
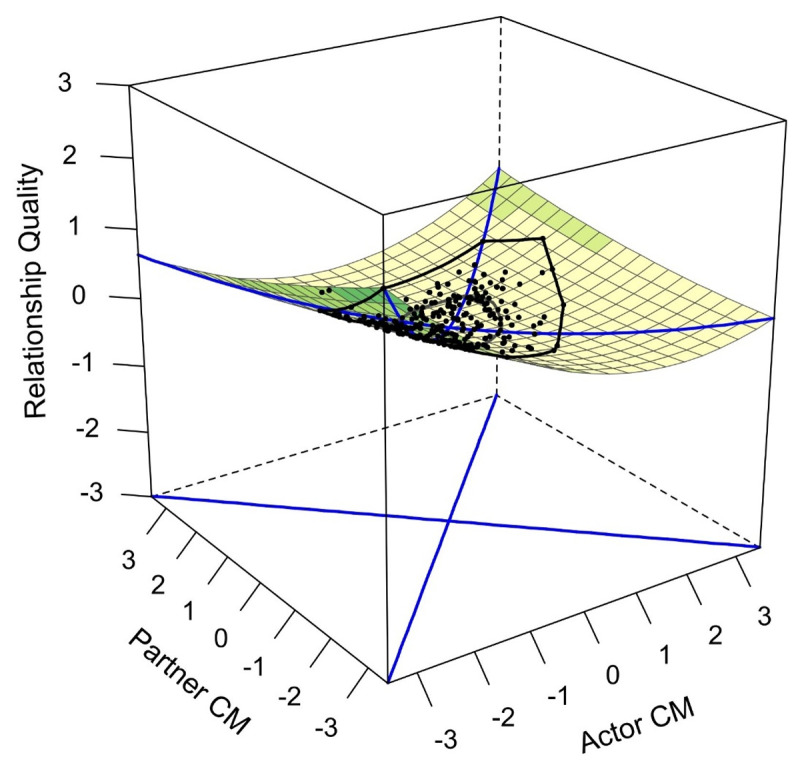
Conspiracy mentality and concurrent relationship quality. *Note. N*_T1_ = 249. CM: Conspiracy mentality.

Regarding conspiracy mentality, the congruence hypothesis was rejected as *a*_4_ was non-significant. Only the surface parameter *a*_1_ was significant (*z* = –2.85, *p* = .004), thus showing a mostly linear negative effect, whereby increasing levels of conspiracy mentality were associated with a decrease in relationship quality. Figure 1 illustrates the relationship between conspiracy mentality and relationship quality.

**Figure 2:**
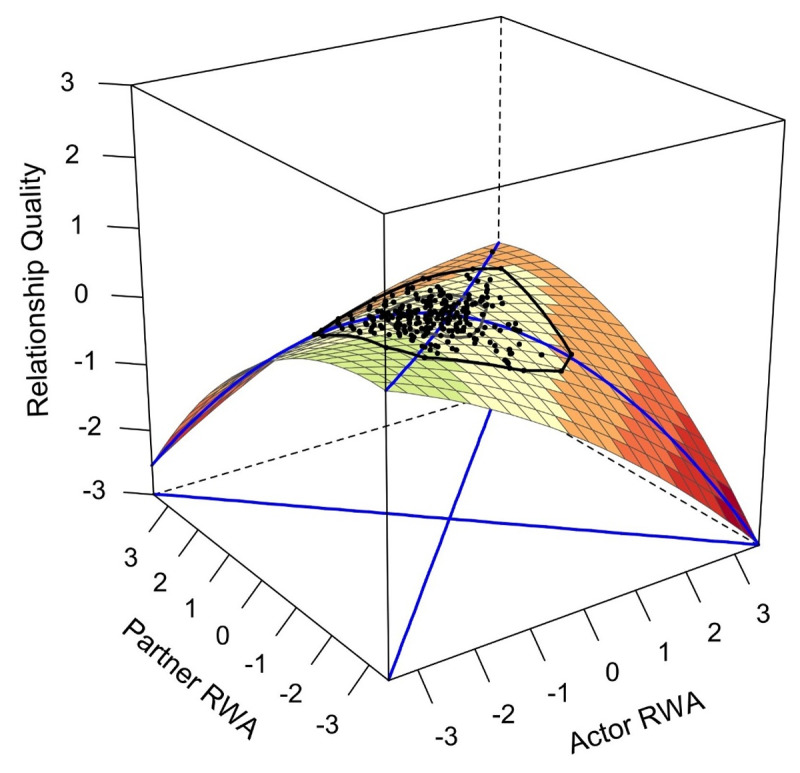
RWA and concurrent relationship quality. *Note. N*_T1_ = 249. RWA: Right-wing authoritarianism.

Analogous to the results for conspiracy mentality, the congruence hypothesis was also rejected for RWA. Although *a*_4_ was negative, it did not reach significance (*z* = –1.58, *p* = .115). The only significant surface parameter was *a*_1_ (*z* = –2.28, *p* = .023), once again indicating a negative linear association of RWA and relationship quality. The resulting response surface plot depicting the relationship between RWA and relationship quality is reported in Figure 2.

In the case of SDO, the congruence hypothesis was also rejected. The only significant surface parameter was again *a*_1_ (*z* = –2.44, *p* = .015), indicating a negative linear association of SDO and relationship quality. The response surface plot for SDO can be found in the accompanying OSF project (OSF E). Taken together, the data did not support a congruence effect of any generalized political attitude on concurrent relationship quality. In an additional exploratory analysis of the observed negative linear main effects for only the subdimension relationship satisfaction, conspiracy mentality retained its negative linear link, while RWA and SDO had a more complex negative curvilinear association (see OSF D6).

**Longitudinal relationship quality (H2b)**. As in the cross-sectional analysis, four DRSAs were computed.^2^ Each DRSA consisted of one of the political attitude dimensions at T1 predicting relationship quality at T2, corrected for relationship quality at T1. For all models, the polynomial regression and DRSA assumptions were completely satisfied. The regression and surface parameters of the DRSAs are presented in Table 5.

**Table 5:** Results of the DRSAs for longitudinal relationship quality.


	CONSPIRACY MENTALITY	RWA	SDO	POLITICAL ORIENTATION

*b*_1_	–0.09 (0.09)	–0.02 (0.10)	–0.03 (0.09)	–0.10 (0.09)

*b*_2_	–0.09 (0.08)	–0.15 (0.11)	–0.08 (0.10)	0.03 (0.10)

*b*_3_	–0.13 (0.09)	–0.14 (0.11)	–0.03 (0.06)	–0.03 (0.06)

*b*_4_	0.33* (0.13)	0.25* (0.12)	0.02 (0.11)	0.04 (0.09)

*b*_5_	0.00 (0.08)	–0.11 (0.08)	0.03 (0.11)	0.01 (0.07)

*a*_1_	–0.18 (0.09)	–0.17 (0.13)	–0.10 (0.14)	–0.08 (0.12)

*a*_2_	0.21* (0.10)	–0.01 (0.11)	0.02 (0.15)	0.01 (0.09)

*a*_3_	0.00 (0.15)	0.14 (0.17)	0.05 (0.13)	–0.13 (0.15)

*a*_4_	–0.45 (0.26)	–0.50* (0.23)	–0.01 (0.22)	–0.06 (0.17)

*a*_5_	–0.13 (0.08)	–0.03 (0.14)	–0.06 (0.10)	0.04 (0.09)


*Note. N*_T2_ = 76. Standard errors in parentheses. Regression estimates can be interpreted as β weights due to prior standardization. *b*_1_: linear actor effect, *b*_2_: linear partner effect, *b*_3_: squared actor effect, *b*_4_: interaction effect, *b*_5_: squared partner effect, *a*_1_ = *b*_1_ + *b*_2_, *a*_2_ = *b*_3_ + *b*_4_ + *b*_5_, *a*_3_ = *b*_1_ – *b*_2_, *a*_4_ = *b*_3_ – *b*_4_ + *b*_5_, *a*_5_ = *b*_3_ – *b*_5_. *p < .05, **p < .01, ***p < .001.

The congruence hypothesis was rejected for conspiracy mentality. Although *a*_4_ was negative, it did not reach significance (*z* = –1.71, *p* = .088). For RWA, the data supported a congruence effect. The surface parameter *a*_4_ was negative and significant (*z* = –2.18, *p* = .029). Fulfilling the other congruence effect criteria, *a*_3_ and *a*_5_ were non-significant. In addition, *a*_1_ and *a*_2_ were also both non-significant, meaning that the data supported a congruence effect in the strict sense for RWA. Finally, the data did not support a congruence effect for SDO. The resulting response surface plots can be found in the accompanying OSF project (OSF E). In an exploratory analysis for the two higher-order value dimensions, we found a congruence effect in the cross-sectional analysis for the dimension self-transcendence vs. self-enhancement (see OSF D8).

**Convergence (H3)**. Although not all of the assumptions were satisfied (see OSF D8), all models were calculated, as linear mixed models are generally robust against violations of their assumptions (Schielzeth et al. 2020). There was no evidence that partners converged in their attitudes over time or that relationship duration negatively moderated this effect. The main effect of the measurement wave was non-significant in all four models. The interaction effect was only significant in the model for RWA, but in the opposite direction as hypothesized (β = 0.24, *t* = 4.69, *p* < .001). The full results of the linear mixed models can be found in the accompanying OSF project (OSF D9).

## Study 2

Study 1 offered important insights into the questions underlying the present research. However, the divergence between the cross-sectional and longitudinal analyses was difficult to interpret, especially in light of the high attrition of the sample between the two measurement time points. Therefore, we decided to repeat the study with a paid sample and longer time difference between the two measurement points. As there was also some divergence between the effects for relationship quality and satisfaction, we decided to focus Study 2 on relationship satisfaction as a dependent variable, as we were most interested in the individual’s subjective happiness with the relationship.

### Methodology

#### Participants and design

The hypotheses and research design of Study 2 were identical to Study 1. Data were collected from December 10 to December 23, 2022 and June 10 to June 23, 2023. A sample of 313 couples was recruited via Prolific and paid £1.80 per person for their participation at T1 and £2.25 per person at T2. One dyad was excluded due to the preregistered careless responder analysis, leaving a final sample size of 312 dyads at T1. The age of the sample ranged from 19 to 85 years (*M* = 38.2 years, *SD* = 10.8 years) and the mean relationship duration was 12.6 years (*SD* = 9.1 years). The final sample size at T2 was 263 dyads.

#### Measures

In contrast to Study 1, the Relationship Assessment Scale (RAS; Hendrick et al. 1998) was used to measure relationship satisfaction. This change was made because the RAS more specifically pertains to our construct of interest, relationship satisfaction, while the previously used RDAS measures multiple constructs and includes only a few items for relationship satisfaction. In addition, the original 40-item Portraits Value Questionnaire (PVQ; Schwartz et al. 2001) was used instead of its short version, the 21-item HVS, in hopes of attaining higher reliability for the value constructs. Otherwise, the measures used were identical to those used in Study 1.

### Results

#### Preparatory analyses

The means, variances, reliabilities, and intercorrelations of all study variables at T1 are reported in Table 6. Analysis of the QQ-plots and descriptive statistics indicated that all study variables were approximately normally distributed. On the individual level, young age, and to a lesser extent, high conspiracy mentality predicted dropout from T1 to T2. On the dyad level, neither the difference scores of the study variables nor the mean dyad relationship satisfaction predicted dropout.

**Table 6:** Means, variances, reliabilities, and intercorrelations of study variables.


	α	*M*	*SD*	1	2	3	4	5	6	7

**1.** CM	.92	4.58	1.12	(.82)						

**2.** RWA	.86	2.65	0.73	.14*	(.79)					

**3.** SDO	.89	2.66	1.22	.13*	.41***	(.82)				

**4.** POL	–	4.59	2.09	.04	.47***	.50***	(.88)			

**5.** ST-SE	.83	1.16	1.25	–.11	–.29***	–.43***	–.30***	(.83)		

**6.** OP-CO	.82	0.15	1.02	.10	–.31***	–.13*	–.24***	–.17***	(.81)	

**7.** RS	.89	5.30	0.79	–.07	–.01	–.11	–.05	.08	.04	(.77)


*Note. N*_T1_ind_ = 624. CM: Conspiracy mentality, RWA: Right-wing authoritarianism, SDO: Social dominance orientation, POL: Political orientation, ST-SE: Self-transcendence vs. self-enhancement, OP-CO: Openness to change vs. conservation, RS: Relationship satisfaction, α: Internal consistency (Cronbach’s alpha). Retest reliabilities are shown on the diagonal (*N*_T2_ind_ = 558). *p < .05, **p < .01, ***p < .001.

#### Hypothesis testing

**Partner Similarity (H1)**. *ICC*s of cross-partner similarity showed that couples displayed similarity above that expected by chance in all political attitude dimensions and both higher-order value dimensions. The *ICC*s, 95% *CI*s, and significance tests are reported in Table 7.

**Table 7:** Cross-partner correlations and significance tests.


	*ICC*	95% *CI*	*F*(311, 312)	*p*

Conspiracy Mentality	.49	[.40, .57]	2.90	<.001

Right-Wing Authoritarianism	.47	[.38, .56]	2.80	<.001

Social Dominance Orientation	.57	[.49, .64]	3.69	<.001

Political Orientation	.66	[.59, .72]	4.83	<.001

Self-Transcendence vs. Self-Enhancement	.25	[.14, .35]	1.66	<.001

Openness to Change vs. Conservation	.36	[.26, .45]	2.12	<.001


*Note. N*_T1_ = 312. *ICC*: Intraclass correlation coefficient, *CI*: Confidence interval. Significance tests (*p* values) were adjusted for multiple comparisons using the Holm-Bonferroni correction.

The cross-partner correlation was larger for generalized political attitudes than for values (all *p*s < .001). Just as in Study 1, correcting the *ICC*s for demographic variables produced only minimal change in their magnitude (see OSF D4). In contrast to Study 1 however, non-uniformity analysis showed that cross-partner similarity was uniform across the full range of all generalized political attitudes (see OSF D5).

**Figure 3:**
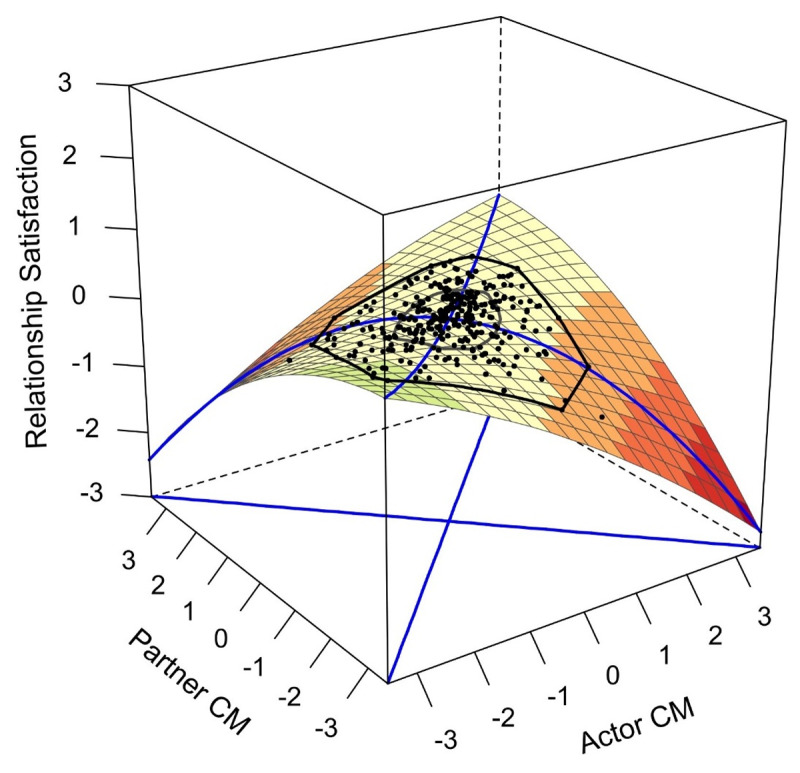
Conspiracy mentality and concurrent relationship satisfaction. *Note. N*_T1_ = 312. CM: Conspiracy mentality.

**Figure 4:**
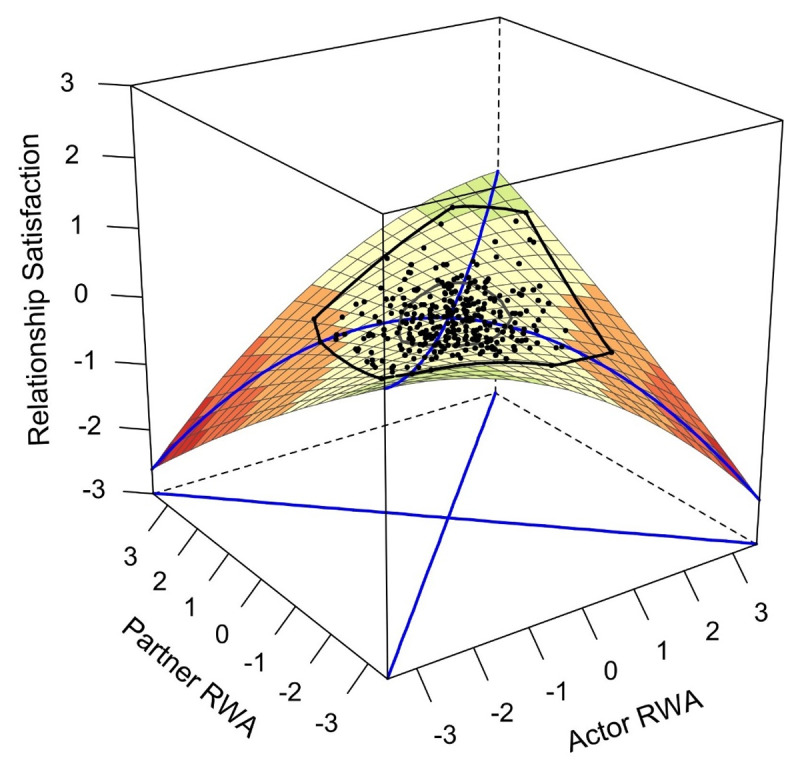
RWA and concurrent relationship satisfaction. *Note. N*_T1_ = 312. RWA: Right-wing authoritarianism.

**Concurrent relationship satisfaction (H2a)**. As in Study 1, four models were computed. The polynomial regression and DRSA assumptions were almost completely satisfied, with the exception of the homoscedasticity assumption for three of the eight regressions. Regarding conspiracy mentality, the congruence hypothesis was rejected, as *a*_4_ was negative, but not significant (*z* = –1.31, *p* = .192). Figure 3 illustrates the relationship between conspiracy mentality and relationship satisfaction. Analogous to the results for conspiracy mentality, the congruence hypothesis was also rejected for RWA, as *a*_4_ also did not reach significance (*z* = –1.54, *p* = .124). The resulting response surface plot depicting the relationship between RWA and relationship satisfaction is reported in Figure 4. In the case of SDO, the congruence hypothesis was also rejected. The only significant surface parameter was *a*_1_ (*z* = –2.24, *p* = .025), indicating a negative linear association of SDO and relationship satisfaction. The regression and surface parameters of the DRSAs are reported in the accompanying OSF project (OSF D10).

**Longitudinal Relationship Satisfaction (H2b)**. As in the cross-sectional analysis, four DRSAs were computed. For all models, the polynomial regression and DRSA assumptions were completely satisfied. The congruence hypothesis was rejected for all generalized political attitudes, as no surface parameters reached significance. The regression and surface parameters as well as the response surface plots for these analyses can be found in the OSF project (OSF D10 & OSF E).

**Convergence (H3)**. There was no evidence that partners converged in their attitudes over time or that relationship duration negatively moderated this effect. Neither the main effect of measurement time point nor the interaction effect between measurement time point and relationship duration was significant in any model (see OSF D9).

## Discussion

### Discussion of Results

This research investigated the dyadic aspects of various highly influential generalized political attitudes. Across two different samples, we found very strong support for the hypothesis of partner similarity in generalized political attitudes, with *ICC*s ranging from .33 (SDO) to .51 (conspiracy mentality) in Study 1 and .47 (RWA) to .57 (SDO) in Study 2. In the context of other homophily research, this level of partner similarity is quite high and comparable to that observed for specific attitudes or education (Watson et al. 2004). This conclusion is also supported by the fact that concordance in generalized political attitudes was at least equal to, and in most cases significantly larger than mean partner similarity in values. Furthermore, the observed similarity did not occur as a byproduct of sorting on demographic traits. This similarity may have implications for the individual, as the romantic partner is among the most frequently named contacts that people discuss political issues with (Beck 1991). Research shows that when individuals have less exposure to differing political views, they are more likely to hold extreme attitudes, more likely to regard opposing viewpoints as illegitimate, less politically tolerant, and less able to present arguments for their own political views (Huckfeldt, Mendez & Osborn 2004; Mutz 2002). Political homophily, especially in the realm of partner relationships, thus has the potential to contribute to political polarization and societal conflicts. As political polarization is often discussed from the perspective of political and (social) media systems, the contribution of individuals self-selecting into politically homogenic romantic relationships has perhaps been underappreciated in the respective literature.

Overall, there was only very little support for the second hypothesis, which stated that higher partner similarity would predict increased relationship quality. Across the different analyses, only RWA displayed patterns akin to a congruence effect, but these rarely reached significance. RWA is likely the most relevant of the tested attitudes for relationship interactions, as it encompasses support for and adherence to traditional norms, which also include attitudes about the organization of and role distribution in relationships. A lack of consensus regarding these issues may decrease relationship quality, but this effect was not found consistently. Interestingly, RWA was also the only attitude displaying some non-uniformity in couple similarity, with individuals high in RWA having more homogenic relationships than those on the low end of the scale. This could reflect the desire for intragroup cohesion, an essential aspect of RWA. Theoretically, this pattern could contribute to differential outcomes in polarization, as individuals high in RWA would have a more limited exposure to diverging opinions than their ideological counterparts. Such asymmetrical polarization, whereby one group moves away from the ideological center at a faster pace than others, has been observed for political parties in the United States (Hacker & Pierson 2015). In addition, both studies found an association of high SDO with lower relationship satisfaction, which may be a consequence of individuals high in SDO favoring competitive over cooperative behavior (Cozzolino & Snyder 2008).

Finally, the data provided no support for the convergence hypothesis in any analysis. Couples did not become more similar over time and individuals in newly formed relationships were just as similar as those in long-established relationships. These findings are in line with previous research on convergence, which has largely been unable to find support for the convergence hypothesis across various domains of partner similarity (Alford et al. 2011; Humbad et al. 2010).

### Causes of Similarity

As shown in this research, there is substantial partner similarity in the domain of generalized political attitudes, but its causes are more difficult to determine. The present research tested several plausible explanations for this phenomenon (demographic byproduct sorting, association with satisfaction, convergence) but did not find strong support for any of them. Although many uncertainties remain, this process of elimination leaves active assortment as a strong candidate as the primary cause of partner similarity in generalized political attitudes. Indeed, there is some evidence that individuals prefer a partner with similar political views (Huber & Malhotra 2017; Watson, Beer & McDade-Montez 2014). Although desired similarity in conspiracy mentality, RWA, and SDO has not yet been subject to investigation, it is plausible to assume that a comparable pattern could emerge. Furthermore, even if individuals are not actively selecting partners based on their ideological attitudes, the influence of generalized political attitudes permeates into everyday life even in situations without apparent political content (Cozzolino & Snyder 2008).

Why would individuals actively choose ideologically similar partners? One possibility is that attitudinal similarity offers psychological benefits by consistently reinforcing one’s own worldviews (Byrne 1971) or improving relationship quality through greater ease of communication and understanding (Anderson, Keltner & John 2003). The present study only found little support for this view, as similarity in generalized political attitudes was mostly unrelated to relationship quality or satisfaction. However, the results of this study do not completely rule out a possible causal influence of attitudinal similarity on relationship quality. In fact, if attitudinal (or any other form of) similarity were to provide psychological benefits, it would be reasonable to expect that individuals who were unable to offer these benefits would need to compensate for this drawback in other ways. Thus, a possible positive effect of similarity could be masked by systematic differences between similar and non-similar relationships. In contrast, it is also possible that individuals actively choose similar partners even though this offers no benefits to them, akin to the information processing perspective (Ajzen 1974). People tend to think that they displayed good judgement in deciding which attitudes to hold and they will think the same of others with similar attitudes. Thus, they could simply prefer others with similar attitudes because they evaluate them more positively. In any case, further research is needed to clarify these possibilities.

### Limitations and future directions

As one limitation of the current research, the temporal distance between the measurement time points (two and six months) was rather short for examining processes such as convergence, changes in relationship satisfaction, and relationship dissolution. Second, both samples had a high mean relationship duration, which made it difficult to examine the role of convergence in the beginning of relationships. Third, there was some non-random attrition in regard to conspiracy mentality across both studies. Fourth, the sample was mainly from western Europe, specifically Germany and the UK, leaving open the possibility of cultural differences in the extent and consequences of ideological homophily. Fifth, although we corrected for some demographic traits such as age and education level, it may also have been interesting to include economic variables such as income, wealth, or economic status. Finally, methodological innovation is moving quickly and more sophisticated RSA-based models such as cubic response surface analysis (Humberg et al. 2022) would allow testing more complex hypotheses such as level-dependent congruence effects. This approach could be especially promising in examining if and how variables differ in the optimal level of congruence depending on their relational implications.

In addition to the ideas discussed as part of the limitations, the present research offers multiple pathways for future studies. Having established that partners exhibit substantial similarity in generalized political attitudes, future research could investigate its origins. As this study obtained no support for most of the proposed alternative explanations, it may be especially worthwhile focusing on the mechanism of active assortment. Such research should establish when and through which specific processes ideological similarity influences relationship formation. To examine these questions, experimental paradigms like the manipulation of fictitious dating profiles or speed-dating events could be employed. Additionally, research could investigate why individuals would prefer to enter a relationship with others who share their ideological attitudes. Does it provide them with psychological benefits or is it simply the result of an information processing heuristic that assumes that others who share our attitudes must be more reasonable people than those who do not? Moreover, future research should be dedicated to the consequences of ideological congruence in relationships on the level of the dyad and society as a whole. For instance, this research could examine whether partner similarity in these dimensions influences relationship outcomes such as relationship dissolution or childrearing results. At societal level, its contribution to political polarization deserves further research attention. Moreover, there may also be reciprocal causal effects, such as growing political polarization causing an increase in partner similarity. Homophily research across other cultures or over longer timeframes could provide further insight into this question. In summary, addressing the context-dependency of the results found in this study would substantially enrich the understanding of ideological homophily.

## Conclusion

Generalized political attitudes such as conspiracy mentality, RWA, and SDO are of immense importance for determining an individual’s political views on a wide range of subjects. However, their role extends far beyond political issues into the most intimate parts of life. As this research demonstrates, individuals do not only sort into like-minded neighborhoods, but also into like-minded relationships. This ideological similarity appeared to be present from the onset of relationships, remained stable over time, and was not reducible to partner concordance in demographic traits, thus suggesting that individual’s generalized political attitudes may fundamentally impact which relationships they enter. The study of social attitudes can only benefit by expanding its scope to dyadic or group processes in order to more closely grasp the complex interactions that lead to the proliferation of political ideologies and conspiracy beliefs throughout society. Hence, this research closes with the hope that it will stimulate further research into this important scientific field.

## Data Accessibility Statement

All data, analysis code, and additional materials have been made available at https://osf.io/d8739.

## Footnotes

https://www.soscisurvey.de.

In the preregistration for Study 1, we reported that we would use the linear regression approach to test for congruence effects if the sample size threshold of 150 dyads was not met. However, even at low sample sizes, the DRSA is the better approach for testing congruence hypotheses. Thus, we report the DRSA result in the text for the sake of consistency. The linear regression results, which are directionally similar, can be found in the accompanying OSF project (OSF D7).
